# A Sustainable Early Warning System Using Rolling Forecasts Based on ANN and Golden Ratio Optimization Methods to Accurately Predict Real-Time Water Levels and Flash Flood

**DOI:** 10.3390/s21134598

**Published:** 2021-07-05

**Authors:** Feras Alasali, Rula Tawalbeh, Zahra Ghanem, Fatima Mohammad, Mohammad Alghazzawi

**Affiliations:** Department of Electrical Engineering, The Hashemite University, Zarqa 13133, Jordan; rulam@hu.edu.jo (R.T.); zahray@hu.edu.jo (Z.G.); 1532077@std.hu.edu.jo (F.M.); 1630516@std.hu.edu.jo (M.A.)

**Keywords:** sustainable monitoring system, real-time early warning, remote sensing, ANN, rolling forecast, flash floods, Jordan

## Abstract

Remote monitoring sensor systems play a significant role in the evaluation and minimization of natural disasters and risk. This article presents a sustainable and real-time early warning system of sensors employed in flash flood prediction by using a rolling forecast model based on Artificial Neural Network (ANN) and Golden Ratio Optimization (GROM) methods. This Early Flood Warning System (EFWS) aims to support decision makers by providing reliable and accurate information and warning about any possible flood events within an efficient lead-time to reduce any damages due to flash floods. In this work, to improve the performance of the EFWS, an ANN forecast model based on a new optimization method, GROM, is developed and compared to the traditional ANN model. Furthermore, due to the lack of literature regarding the optimal ANN structural model for forecasting the flash flood, this paper is one of the first extensive investigations into the impact of using different exogenous variables and parameters on the ANN structure. The effect of using a rolling forecast model compared to fixed model on the accuracy of the forecasts is investigated as well. The results indicate that the rolling ANN forecast model based on GROM successfully improved the model accuracy by 40% compared to the traditional ANN model and by 93.5% compared to the fixed forecast model.

## 1. Introduction

### 1.1. Background

Worldwide, climate change, with its related phenomena such as flash floods, has shown high negative impacts on both environmental and human society [1]. The flash flood problem is a natural hazard and one of the most frequent events, which is caused mainly by heavy rains within a short duration of time (heavy rainfall) and the limited water drainage infrastructure [1,2,3]. Therefore, the flash flood events are related to the frequency of rainfall, the properties of drainage basins, the physical properties of the land, the land use and cover characteristics [3,4]. For example, the increase in urbanization with limited spaces to absorb and hold rainwater will increase the risk and possibility of flash floods. Globally, floods disasters which occurred between 2001 and 2018 were responsible for 504 billion USD financial losses and a 94,000 death toll [5].

In recent years, many areas in Jordan, in particular downtown/Amman, have experienced flash flood events due to the city’s nature and climate change [6]. These flood events caused a loss in human lives, properties (roads and agricultural areas), and economic loss. Most of the flash flood events in the downtown/Amman area are caused by a combination of the geographical nature of this area, heavy rainfall and the limited drainage systems [6,7]. The downtown area in Jordan is surrounded by Amman’s mountains. Therefore, it is considered as an outlet for water flow from all over the city, which increases the probability of flash flood events [7].

Recently, flash flood events occurred in the downtown/Amman area (2018, 2019 and 2020). These events were characterized by heavy rainfall within a short duration, flash floods caused death toll, properties and infrastructure damages and economic loss. According to a governmental study [8], the flash flood that happened in February 2019 has mainly occurred due to the limitation of water drainage systems such as the size, efficiency and quality of the system to handle the rainfall. This event was characterized by 658 $m^{3}$/s of rainfall within two hours and considered as a flash flood for the downtown/Amman area, in particular Quraish Street in ‘Saqf Al-sail’, and caused five million Jordan Dinars lost by destroying approximately 200 shops in this street. In another event, January 2020, the rainfall caused floods in downtown/Amman and the 7th Circle areas destroying around 100 stores [9]. The two mentioned floods as well as other floods were not only responsible for store losses but also for life casualties and property destruction. The heavy rainfall events with limited drainage systems and unplanned urban expansion caused flash floods in other regions of Jordan, such as Irbid and Al Mafraq cities, similar to the downtown/Amman area. In addition, one of the most dramatic disasters happened in the Dead Sea/Alzarqa-Maeen valley, on the 25 October 2018, when a flash flood caused death toll of 21 young children during a school trip.

Jordan faces significant challenges in developing and implementing sustainable plans to handle flash flood problems due to the limitation of infrastructure and water resources. There is a limited number of research [10,11,12] that have addressed the flash flood problems in Jordan, and they concentrate on the Petra area (desert valley area). To the author’s knowledge, no studies have discussed the flash flood in the cities of Jordan. Although there is a high demand due to climate change for understanding flash floods in cities, and the discussions about strategies to handle floods in cities have been more significant [13], there is a lack of developing sustainable plans to face flash floods risk. A flash flood early warning system is an effective fundamental tool to minimize damages and loss due to floods. On the other hand, a reliable forecasting model for flash floods is significant to protect human lives, minimize the flood damage and develop a reliable flash flood early warning system. This project introduces a warning system called the Early Flood Warning System (EFWS) to handle the floods problems in the cities of Jordan. The proposed system will be developed based on two main parameters. Firstly, real-time water level measurements through an ultrasonic sensor, located in the street water drainage. Secondly, a rolling flood forecasting model based Artificial Neural Network (ANN) to estimate the water level in the water drainage systems during the storm period and improve the early warning system performance.

### 1.2. Literature Review

The United Nations (UN) defined the early warning system as a model that generates timely and meaningful warning messages to help governments, individuals and communities to handle possible risks and help to reduce the possibility of damages or loss [5]. In the last decade, flooding disaster events caused serious economic and human losses. Therefore, the importance of having early warnings for flash floods has increased in order to avoid or minimize the negative impacts of flooding.

EFWSs have been developed and implemented around the world with different structures. The basic composition of an EFWS that ensures high functionality was described and reviewed in [13,14]. However, the EFWS introduced in the literature focused on systems suitable for rivers and valley areas and does not present a system suitable for cities. For example, in Kenya, an EFWS was introduced to reduce flash flood disaster risks and losses [15]. The proposed system by [15] used microwave communication links and a geostationary satellite system to collect rainfall information, which was analyzed to distribute warning messages through the mobile phone operator networks. In Belgium [16] a weather forecasting model was presented based on an ensemble numerical method and collected data from the rainfall radar station to estimate the future rainfall and issue warning alarms. Apollonio et al. [17] presented the benefits of using a real-time measurement system for rainfall to identify hazard areas and create an estimating flood risk map. Most researches have focused on developing EFWS using rainfall-runoff models and considering regional parameters. For example, a wireless sensor network model was developed in Colombia to measure weather parameters such as temperature and humidity [18]. The weather information will be analyzed to select the probability of having heavy rainfall. Similarly, in another study [19], a network of wireless sensors was utilized in an EFWS to collect hydrological data (water level and precipitation) and then use it for flood event analysis and simulation. The system was used to predict water-level reaching time and the level of hazard and to specify endangered areas for regions in Saudi Arabia. Another EFWS was developed in Florida (USA) based on using ultrasonic water level sensors and cameras to provide a data processing module, where this has the necessary information to determine the warning [20]. The water level sensors based on radar technology were used to develop an EFWS in Bonn/Germany [21]. The water level sends the real-time measurements to the fire brigade, and then the warning will be issued in case the water level exceeded the specified threshold value [20,21,22]. Overall, the previous models presented in the literature do not take into account the weather forecast error impacts on the EFWS and vulnerability of infrastructure in cities. In addition, there is a lack of developing an accurate and effective EFWS to predict flash floods. The machine learning algorithms have been used in [23] to solve energy consumption problems and find the wireless network structure. In another work, the machine learning algorithms have been employed to present a smart city proposal based on an intelligent transportation system [24].

Recently, researchers tend to use new artificial techniques such as ANN to predict the flood events for rivers areas based on the water levels of rivers [22] and different complex applications [23,24]. Wu et al. [25] and Aichouri et al. [26] developed an ANN model to estimate the variations in water level of rivers, and they proposed linear regression and nonlinear equations, respectively. In order to improve the prediction accuracy, Zhang et al. [27] developed an Elman ANN based on considering the previous and current time measurements. For forecasting stochastic events such as flash flood, which is a challenging task, new intelligence and new techniques are required [26,27]. The ANN has been used in the literature to predict the water level and focus on increasing the reliability of the EFWS [22]. However, there is a lack of investigation on the ANN structural model, which will help to optimally select the ANN structure (input, hidden, and output layers). One of the objectives in this study is to develop a novel rolling EFWS for flash floods in streets (drainage system) using real-time measurement based on ultrasonic sensors and the ANN model. In the literature [22,27], the traditional and common optimization methods—namely, steepest descent and gauss newton methods—are employed in ANN as a learning algorithm and guarantee optimal performance. In general, these traditional algorithms are efficiently limited in solving high level of uncertainties or stochastic application nature. Recently, new intelligent optimization techniques such as the Recurrent Neural Network (RNN) and the Golden Ratio Optimization Method (GROM) have been employed in ANN to forecast the stochastic energy demand applications such as renewable energy [25]. The GROM as a modern learning algorithm aims to improve the ANN training and validation process by minimizing the tuning time to achieve the global optimal solution compared to other traditional optimization methods in ANN such as gradient descent training algorithm [25]. Therefore, it is important to examine new optimization algorithms such as the GROM technique to efficiently handle the uncertainties in EFWS such as weather and water flow data and increase the forecast accuracy. In this article, the GROM algorithm is used as a new metaheuristic optimizer to train the ANN to chive global parameters and weights within a minimum computational time.

### 1.3. Contributions

The previous literature has highlighted the flash flood disaster and introduced early warning systems as a significant tool to avoid and reduce flash flood risks. However, the presented early warning systems in the literature focused on using sensors system to check the water level without taking into account the weather forecast data [19,20,21] or using the weather forecast data to estimate the rainfall and issue a warning [24,25,26]. In general, the EFWS presented by the literature [19,20,21,22] aimed to cover rivers areas and there is a lack of developing an accurate EFWS to predict flash floods in streets or cities. Jordan faced and will face many local flash flooding events such as those that occurred in downtown/Amman which caused economic damages and human losses. Thus, the key contributions of this article are listed as follows:

Developing an accurate and efficient EFWS for floods in the street by using real-time monitoring system and a new forecast model. The proposed EFWS aims to support the community and decision-makers by providing reliable and accurate information and warning through different ways about any possible flood events within an efficient lead-time to reduce any damages due to flash floods

- Developing a novel rolling forecast model for flash-floods-based ANN technique equipped with an early warning system based on the weather forecast data and real-time measurements of water level in the street drainage system (downtown/Amman). The proposed EFWS in this paper aims to support decision makers by providing them with reliable and accurate information and warning about any possible flood events. Furthermore, the EFWS will provide accurate forecasts with efficient lead-time to reduce any damages due to flash floods.
- This paper develops an ANN using a new optimization method, called the Golden Ratio Optimization Method (GROM) technique, to estimate the water level over one day ahead during flash flood events. The result of the new forecast model (ANN based on GROM) will be compared to the literature, the traditional ANN model.
- This article examines the impact of using different exogenous variables on the ANN forecast and investigate the optimal ANN structure for forecasting the flash flood in Jordan.
- Developing a rolling ANN forecast model to treat the stochasticity of weather forecast data and improve the prediction accuracy of the flash flood in Jordan compared to fixed point forecast models.

### 1.4. Outline of Paper

The remaining sections of this paper are organized as follows. In Section 2, the methodology of the EFWS and system modeling are presented. Section 3 introduces the EFWS system modeling and study area. The rolling ANN Forecast Model Optimized by GROM is presented in Section 4. Then, the EFWS results are shown and discussed in Section 5. Finally, the summary and conclusions are presented in Section 6.

## 2. Methodology

**Figure 1:**
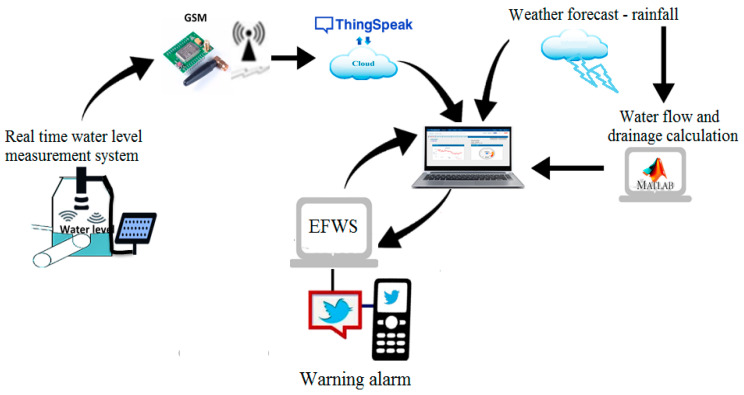
The key elements of the EFWS.

A flash flood is a sudden event that cause economic damages and human loss. In order to minimize the risks caused by flash floods, early warning systems are required. To design a suitable early warning system, it is necessary to understand weather information, hazard monitoring and disaster risk assessment [13,16,17]. In this project, the EFWS integrates many subsystems (or elements) that include a real-time water level measurement system, a communication system, water flow and level estimation model based on the weather forecast data, forecasting flash flood model and hazard monitoring model as shown in Figure 1. Flash flooding estimation is subject to a wide range of variables such as weather information and water sector infrastructures. Therefore, the warning system needs to include a forecast system that handles a range of potential events. The actual measurements and updated information from the site will be used as feedback to update the forecast and warning model. As shown in Figure 1, the project proposed model includes a real-time monitoring system for water level using an ultrasonic sensor powered by a PV system. The real-time monitoring system will be mounted on the top of the water drain. Here, the GSM technology will be used to send and receive the data from the monitoring system to the PC workstation (as a control station).

**Figure 2:**
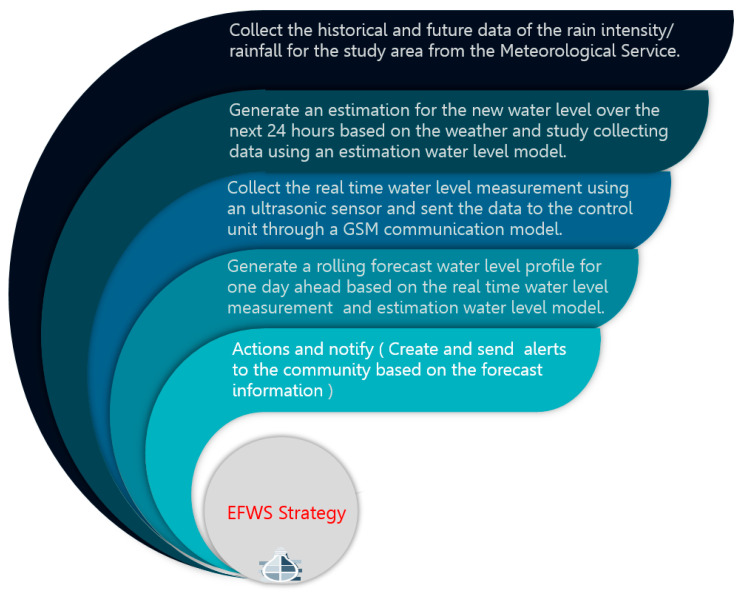
The basic strategy principle of EFWS.

The proposed design in this project aims to increase the awareness time before flooding occurs to reduce disaster risks. Flash flooding estimation is subject to a wide range of variables such as weather information and water sector infrastructures. Therefore, the warning system needs to include a forecast system that handles a range of potential events. However, the weather and flash flood forecasts have a high level of uncertainties which increase the challenge of developing an accurate flood forecast profile. The main aim of the proposed EFWS concept is to develop a flood warning system that is taking both the real-time water level measurements as well as the vulnerability of infrastructure (drainage system) by calculating the estimation of water level based on weather forecast data. The basic strategy principle of the proposed EFWS is introduced in Figure 2. Firstly, the collected available data about the infrastructure of the area from the government side (depth, diameter and roughness coefficient) and channel slope data are used to find the hydraulic and hydrologic analysis and calculate the water drainage flow and maximum discharge of the drainage channel using Manning formula method. Then, the weather forecast data and the level of water from sensors are the main requirements for a system that generates an early warning plan and a flash flood forecast that will be provided for days ahead. In the next stage, the workstation receives the updated water level and flash flood prediction data. These data are used to generate a warning plan for days ahead. Furthermore, the warning plan will be updated at every time step. This process aims to minimize the impact of the forecast error on the warning system and avoid an unnecessary or misleading warning plan. The warning plan is then evaluated based on a risk analysis tool to generate an emergency plan or publish awareness. The actual measurements and updated information from the site will be used as feedback to update the forecast and warning model. The main goal of the project is to come up with a flash flood forecast and warning model to minimize the risks and losses of flash floods by using weather forecast and real-time measurement data.

## 3. EFWS System Modeling and Study Area

**Figure 3:**
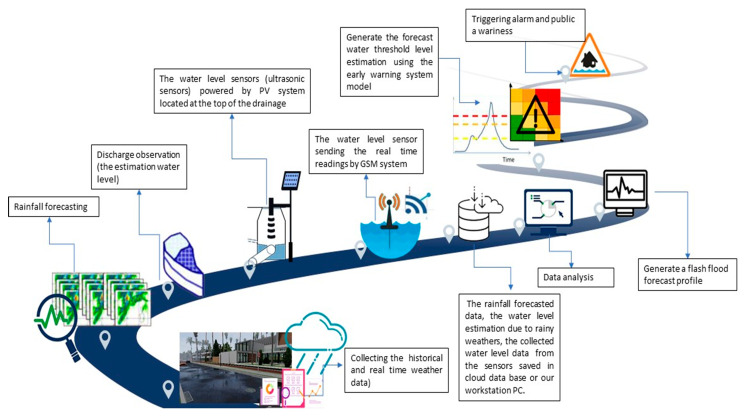
The main mechanism of the EFWS.

**Figure 4:**
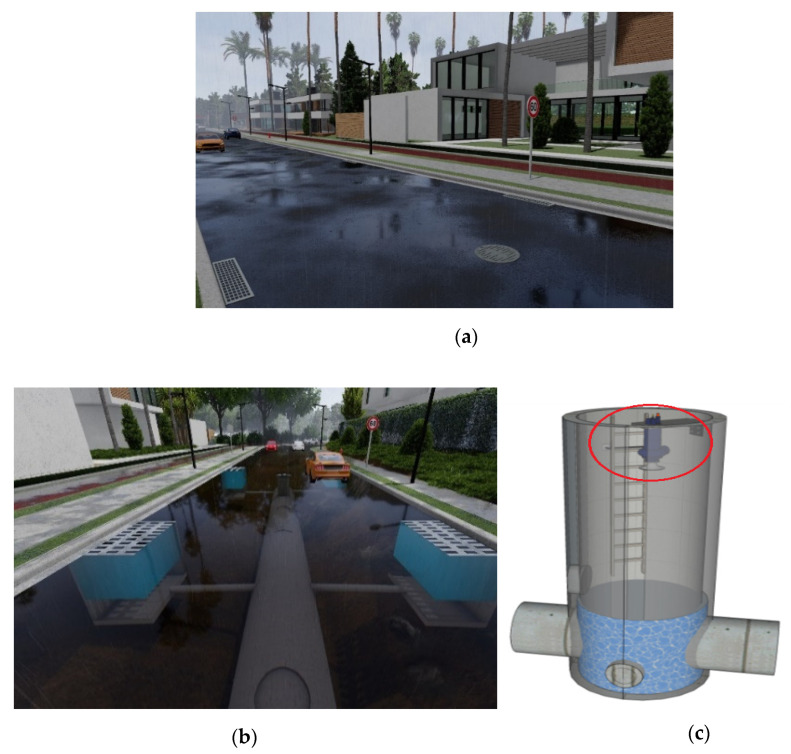
(**a**) Simulation for a street area with the drainage system. (**b**) Simulation for the drainage system in the street. (**c**) The real-time measuring system (ultrasonic sensor) located at the top of the water drainage system.

EFWS in this study is an adaptive rolling forecasting and monitoring system that is based on real-time measurements and future weather condition, taking into account the vulnerability of the drainage system in the study area. This compensation helps the EFWS model to be more accurate and reliable compared to the traditional warning model. Figure 3 presents the main mechanism of the flood forecasting and early warning system (EFWS). The real-time measurements and updated information from the site will be used as feedback to update the forecast and warning model. As shown in Figure 3, the proposed EFWS model includes a real-time monitoring system to measure the water level in the drainage system using an ultrasonic sensor powered by a PV system as a sustainable solution. The real-time monitoring system will be mounted on the top of the water drainage system, as shown in Figure 4. Here, the GPRS/3G/4G communication modules will be used to send and receive the data from the monitoring system to the PC workstation (as a control station). On the other hand, the weather forecast data (rainfall/rain intensity) and drainage system data will be used to calculate and estimate the new water level during flash flood events. To present the drainage system characteristics and calculate the water drainage flow and discharge, the Bentley FlowMaster computer program was used. The output data (water drainage flow and discharge) and weather forecast data (rainfall/rain intensity) will be fed to a Matlab model developed in this study to calculate and estimate the water level based on the rational method equation [2,19], further details in Section 3.1. Finally, both data (real-time measurements and future water level estimation) will be used to feed the rolling forecast model, in Section 4, and issue a warning alarm if necessary.

### 3.1. Study Area and the Water Drainage System

**Figure 5:**
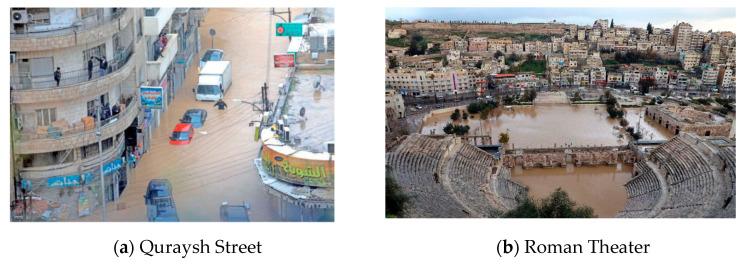
Downtown/Amman floods, February 2019.

The downtown area (Al-Balad) is the oldest part of the capital city of Jordan, Amman, at latitude 31°57′24.1″ N and longitude 35°56′42.0″ E. It represents the heart of Amman, where the primary shops are located and many events take place. Geologically, the downtown area is located 700 m above the sea and surrounded by a number of mountains [6,7]. The weather in this area is almost hot in summer with average temperatures of 30 °C and cold in winter with 5 °C as average temperature. The average annual precipitation in the downtown area is between 250 to 350 mm. In general, around 90% of the total annual precipitation happens over four months (December–March) [8,9]. Over the last three years (2018, 2019 and 2020), flash floods events occurred in the downtown/Amman area and caused death toll, properties and infrastructure damages and economic loss, as seen in Figure 5. According to a governmental study [8], the flash flood in downtown that happened on February 2019 has mainly occurred due to the limitation of water drainage systems such as the size, efficiency and quality of the system to handle the rainfall.

**Figure 6:**
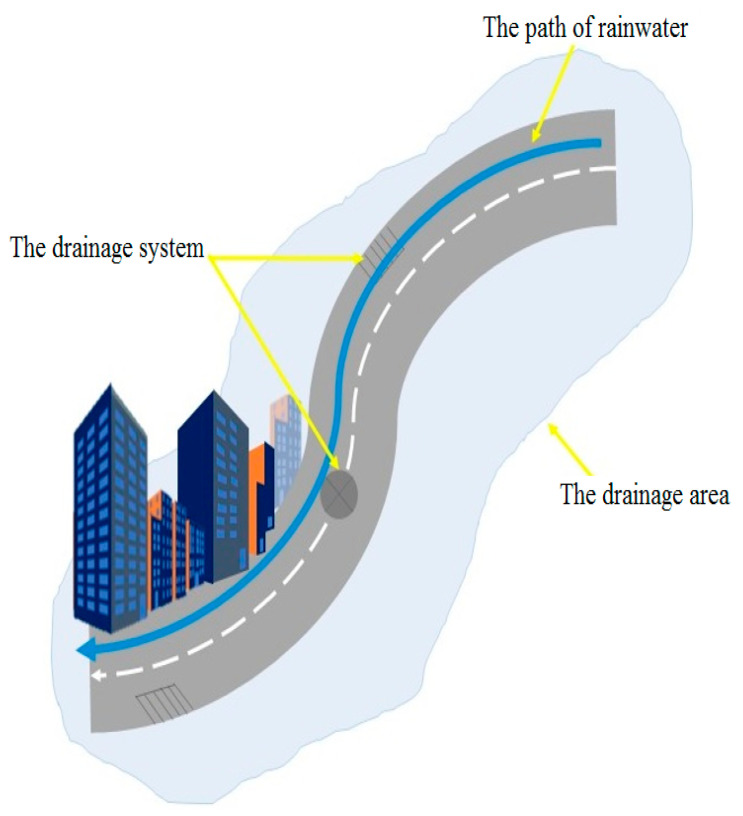
The rainfall and drainage system area.

**Figure 7:**
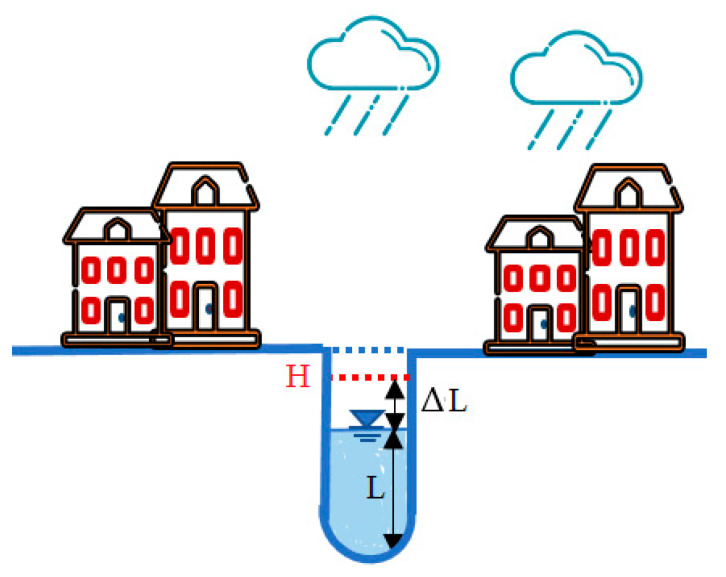
The water level analysis in the drainage system.

As previously mentioned, the downtown area has a vulnerability of infrastructure (water and stream drainage system) and a lack of information and data. Therefore, for the hydraulic and hydrologic analysis, we firstly used Google Earth Pro simulation to estimate the channel slope of the stream drainage system. In this study, due to the lack of information related to the infrastructure such as the water pipe slope in the water drainage system, the slope of streets is used to estimate the water pipe slopes. Here, the Google Earth Pro simulations are used to find and determine the slope of streets. Then, the collected available data about the infrastructure of the area from the government side (depth, diameter and roughness coefficient) and channel slope data is used as input data for the most common program in hydraulic and hydrologic engineering called Bentley FlowMaster software. This software has been used here to calculate the water drainage flow and maximum discharge of the drainage channel using the Manning formula method [2,3,17,18,19]. As shown in Figure 6, to estimate the adding water level in the drainage system due to rainfall events, it is important to know firstly the discharge rate of the drainage system (which is previously completed using Bentley FlowMaster software). Secondly, we need to estimate the new water flow and level coining from the rainfall event. The weather forecast data (rainfall/rain intensity) and area information (the path of rainwater, drainage area) are used to calculate and estimate the water level rise based on the rational method equation [2,3,17], and for this purpose, a Matlab program has been developed in this paper. The EFWS process is based on two main points: the current water level, *L*, which will be measured on real-time basis using the ultrasonic sensor model, as described in Section 3.2, and the estimation of the water level rise, Δ*L*, as shown in Figure 7. The Δ*L* is the difference between the water level rise due to rain and the channel discharge rate. In Figure 7, the estimated new level of water in the drainage system, *H*, where H=L+ΔL, will be used to issue a flood warning if it reached the threshold water level. The warning model will be discussed in Section 3.3.

### 3.2. Real-Time Measurement Model

**Figure 8:**
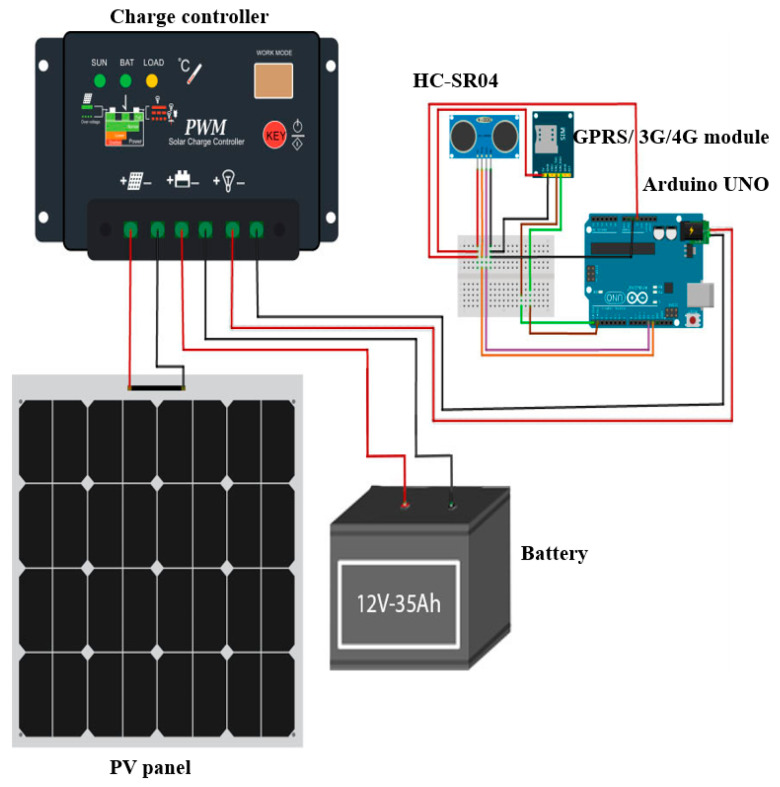
The circuit and component of the real-time measurement model.

**Figure 9:**
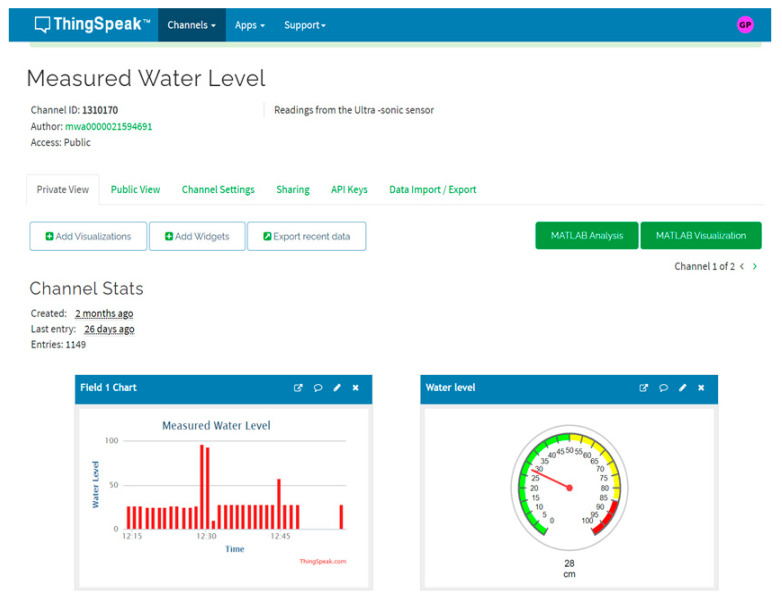
An example database from the ultrasonic sensor with the Arduino system via ThingSpeak cloud.

In order to measure the water level in the drainage system, *L*, a real-time monitoring system has been designed and developed in this study. As shown in Figure 8, the real-time measurement model consists of an ultrasonic sensor, Arduino micro-controller, GPRS/3G/4G communication module, solar panel, battery and charge controller. The ultrasonic sensor is used to measure the water level which is controlled by Arduino micro-controller, which in turn sends the data through the communication module to the workstation via ThingSpeak Cloud, as shown in Figure 9. The real-time measurement data will be received by the workstation through the Matlab program, which has been already developed for this purpose, as previously discussed in Section 3.1. In this real-time system, the solar panel, battery and charge controller is used to provide the required power to operate and run the real measurement model. The size and specification of the solar panel, battery and charge controller system has been calculated and determined to provide a reliable and stable system that can provide the required energy to the system during nights and cloudy winter conditions (with two days of absence of sun). The main equipment specifications of the time measurement model are presented in Table 1. The ultrasonic sensor (HC-SR04) has been chosen due to its ability to accurately work in dark environments, wide distance range and high measuring accuracy compared to other type of sensors such as IR sensor and waterproof sensor JSN-SR04T sensor [28].

### 3.3. Flood Warning System

The Yokohama Strategy and the Sendai Framework for risk assessment [13,20] introduce the significance of efficient EFWS in reducing disaster risk. In general, EFWS needs to generate meaningful and timely warnings to the government and communities before and during flood events. This is will help the government and communities to act safely and correctly to minimize the damages [13,20]. Therefore, it is important to develop an accurate flood forecast model based on the real-time water level measurements, and the estimation of water level rise, Δ*L*. The water level in the drainage system, *L*, undergoes variations over time due to weather, infrastructure and human factors. For this reason, the estimating of the new water level, *H*, needs to be a continuous process that helps to create an accurate and reliable warning alarm. The design of the warning alarm model in the EFWS for the drainage channel, as discussed in Section 3.1, is obtained by estimating the maximum water level for the next 24 h. The rolling forecast model for the water level over one day ahead using ANN will be discussed in details in Section 4.

**Figure 10:**
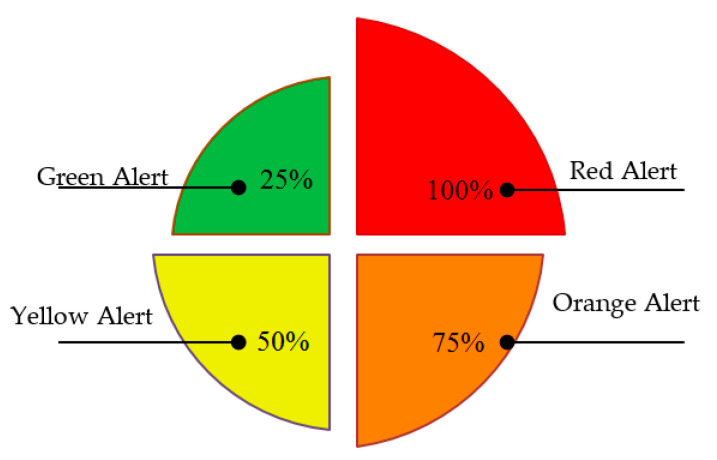
The warning alarm level.

**Figure 11:**
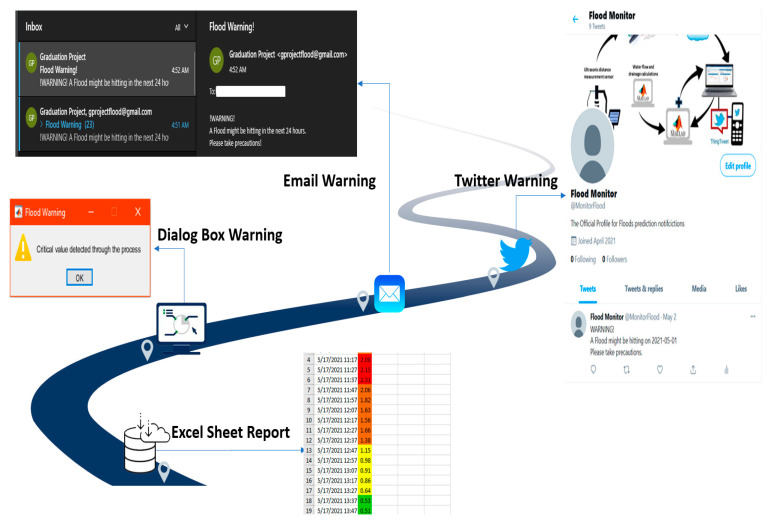
The warning alarm notifications.

The warning model is developed based on thresholds values of the water level in the drainage channel. A method based on the rolling forecast results of the water level is used, the thresholds of water level were set as shown in Figure 10. The green level is when *H* is at less than 25% of the capacity of the drainage channel, the yellow level is between 25% and 50% of the capacity, the orange level is between 50% and 75% of the capacity and the red level if it exceeds 75% of its capacity. A day ahead alert will be issued in the EFWS and every time step the alert level changes state. In this study, the proposed EFWS will generate different type of notifications if the water level estimated in the orange and red levels. As shown in Figure 11, the proposed EFWS model will generate daily and hourly reports (excel sheets) and dialog box warning (lights and sound) in the workstation (control center). This data and information will be available for the decision maker and government to take necessary decision and actions. In addition, the proposed EFWS model will send an email notification to user and post a warning message on Twitter if the estimated water level is in orange or red zones. The notifications and EFWS reports will automatically change according to the updated water level estimation. In this project, a day-ahead warning plan will be issued and updated every 10 min based on real-time measurements. The rolling forecast and data collection and processing will be discussed in detail in the next section. In general, the time between the alert and potential flash with a minimum value equal to 10 min is enough for people to exit the risk zone.

## 4. Rolling ANN Forecast Model Optimized by GROM

**Figure 12:**
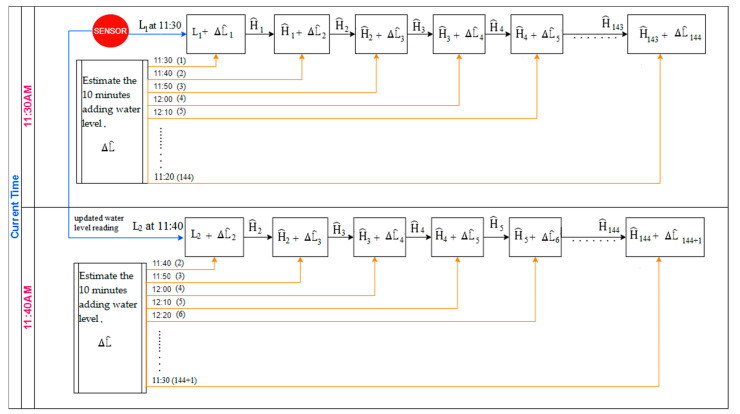
The rolling forecast model in EFWS.

In the literature, the forecasting process in EFWS is designed to use weather and infrastructure to estimate the water level based on rolling and fixed forecast models. However, in realistic scenarios, the water level during storm conditions or flash flood events has a high level of uncertainty. Accurate forecast models with the ability to handle these uncertainties are required. Here and unlike the previous works, a rolling forecast model has been designed and developed based on the ANN technique. The rolling forecast process aims to firstly generate a water level prediction profile for one day ahead with 10 min time resolution, and then the model will be updated at every time step by using the real-time measurement and forecast error. The rolling process will help to minimize the impact uncertainties and improve the forecast model accuracy compared to a fixed forecast model. As shown in Figure 12, the flash forecast model is designed to predict the water level, $\hat{H}\left(t\right),$ for the next day with 10 min time resolution, where t represents the current time step and (ˆ) notation is for forecasting data. The flash flood forecasting model in this paper based on ANN will create a rolling water level forecast as shown in Table 2 and Figure 12 [29,30].

### 4.1. ANN Forecast Model for ESWS Optimized by GROM

Generally, the prediction of flash flood events or water levels during storm conditions is a challenging and complex problem, consisting of several nonlinear relationships such as weather status, infrastructure and human factors. A wide range of ANN techniques and structures are used to predict and estimate different applications such as energy and control model due to their flexibility and ability to solve complex and nonlinear relationships [30,31,32]. Recently, researchers tend to use ANN to predict the flash flood events for river areas based on the water levels of rivers [25,26,27]. However, there is a lack of investigation on the ANN structural model, a model that will help to optimally select the ANN structure (input, hidden, and output layers). One of the objectives of this study is to develop a novel rolling EFWS for flash floods in streets (drainage system) using real-time measurement based on ultrasonic sensors and the ANN model. In addition, this article aims to examine the impact of using different exogenous variables on the ANN forecast and investigate the optimal ANN structural model for forecasting the flash flood in Jordan.

**Figure 13:**
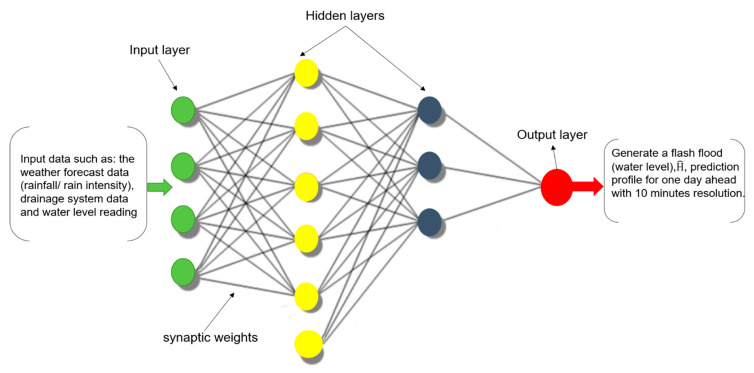
The basic structure of the ANN forecast model for EFWS.

An artificial neuron model mimics how biological NNs of the central nervous system operate and learn. The mathematical model of ANN is constructed by multiple layers (input, hidden and output) of neurons which are interconnected by synaptic weights from each neuron in one layer to each neuron in the next layer, as shown in Figure 13. In the standard structure of an ANN model, the output of each individual neuron will represent the output of the previous layer neurons (as input signal) multiplied by synapse weights and modified by an activation function [24]. The activation function aims to modify the summation of input signals in the neuron before being output by using a nonlinear function such as a sigmoid and tangent (tanh) functions. The output of each neuron, as shown in Figure 13, can be mathematically represented in Equations (1) and (2):

(1) $$H_{i}=F\left(\overset{m}{\underset{j=1}{\sum }}W_{ij}X_{j}\right)$$

(2) $$F\left(Q\right)=\frac{1}{1+e^{-Q}}$$

where $H_{i}$ is the summation of synaptic weights $W_{ij}$ (between the input neuron j and the hidden neuron (i) multiplied by the outputs of each individual neuron $X_{j}$ (from the previous layer) and m is the number of neurons from the previous layer which are connected to the neuron i. In the structure of a typical individual artificial neuron, the summation of input signals from several synapses, as seen in Figure 13, are transferred through an activation function F for hidden layers described by Equation (2) for the sigmoid activation function, the most common nonlinear activation function, where $Q=\overset{m}{\underset{j=1}{\sum }}W_{ij}X_{j}$. The majority of the current ANN models use sigmoid functions, due to its differentiability which make it computationally easy to calculate. The main aim of including activation functions as scalar functions in the ANN structure is to introduce the nonlinearity into the prediction model, which helps it to solve nonlinear and complex problems and limit the neuron output [24,30]. To forecast the water level in EFWS over one day ahead, two ANN models are used:

- Traditional ANN model: this proposed model is a feedforward ANN model trained by one of the most common methods called Levenberg-Marquard and the neuron activated by a sigmoid function. This traditional ANN model has been widely used in the literature with stochastic targets [24].
- ANN forecast model optimized by Golden Ratio Optimization Method (GROM) algorithm [33]: the feedforward ANN model will use the GROM algorithm to handle the learning process and achieve the optimal performance in ANN.

#### 4.1.1. Implementation of ANN Models (Traditional and GROM Models)

As previously discussed, there is a lack of investigation into the ANN structural model for flash flood or water level forecasting events to optimally determine the ANN structure (input, hidden, and output layers). In general, there is no favorite approach or method to select and guarantee the best or optimal number of layers or neurons. The ANN structure depends on the availability of data, data resolution, correlations between the inputs and outputs and complexity of the problem. For example, increasing the number of neurons and hidden layers can lead to an overfitted situation and can increase the computational cost (the required time to run the model) [24,25]. The literature showed that one to three hidden layers with less than 20 neurons at each layer can be sufficient to achieve the optimal results without leading to overfitting in the ANN for different applications. In this paper, experiments were carried out to investigate the optimal ANN structure for EFWS that generate the minimum forecast error based on a different number of hidden layers (1 to 5) and a different number of hidden neurons (10 to 50) [30,31]. The ANN structure is developed and designed by selecting the main ANN parameters models, as follows:

- Inputs variables: initially, the following variables (adding water level at a time, $\Delta \hat{L}$ (*t*), and real-time measurement for the water level, L (t−10) have been carefully selected as the main input variables, where they are directly connected to calculate the future water level. In order to improve the forecast model performance and select more suitable input variables, the previous time steps from 10 min until one hour, the previous day water level at the same time, previous day data of daily rain intensity and temperature are tested in this work as external variables.
- Output variables: the future water level, $\hat{H}\left(t\right),$ over the next day with 10 min time resolution.
- Data processing: weather conditions data has been collected over three years from 2017 to 2019. These data sets are divided into training (60%), validation (10%) and testing data sets (30%). In general, the training set is the largest data set, and it is used to fit the parameters of the model, train the forecast model and find the patterns. The training set needs to be large enough to represent the data characteristics. This data set is mainly used to select the potential models. Then the validation set is used to try and find the best of these by training each of the model parameters in the training set and then testing the errors for forecasts in the validation set. The validation set is used as a final performance check for the trained network before testing the model with around 10% of the training set. The reason for this structure is to avoid a specific good forecast on the training set which turns out to be inaccurate in the test set. In addition, this way aims to avoid “overfitting” by selecting a forecast that is over-trained on the training set. Finally, the testing set is used to provide an unbiased evaluation of the final forecast model and is typically 10% to 30% of the training set.
- The number of hidden layers and neurons: to optimally select the number of hidden layers and neurons for the ANN in EFW, different number of hidden layers (1 to 5) and different number of hidden neurons (10 to 50) has been tested.
- Training and learning algorithm: Levenberg-Marquardt and GROM algorithms are used and tested.
- The stopping criteria: in case there are no improvements in the accuracy.

The ANN forecasts for EFWS application are evaluated for different parameters and structures in order to determine the optimal and suitable model parameters. The impact of the number of hidden layers and neurons and different input variables on the forecast model accuracy are examined. In the following section, Section 5, the analysis and results of each parameter will be presented.

#### 4.1.2. ANN Forecast Model Based on GROM Algorithm

In the literature, the traditional ANN forecast model is developed and designed based on using a number of traditional optimization techniques such as gauss newton and gradient descent methods. The Levenberg-Marquardt with back-propagation algorithm modifies the weights by calculating the error in a backward order, from the output layer to the input layer. In general, the ANNs use the training set to learn the key patterns between the input and output information by suitable synaptic weights. When each pattern is read, and the training reaches a satisfactory level, the ANN holds the synaptic weights and uses the trained network to predict the future or make decisions. The network trains over a range of patterns, by using the back-propagation algorithm. This training process aims to improve the performance of the ANN model by minimizing the total squared error, Err, as described by Equation (3). Furthermore, the training process will stop when there is no further reduction in the error function.

(3) $$Err=\frac{1}{2}\;\;\;\overset{K}{\underset{k=1}{\sum }}\underset{i}{\sum }{\left|H_{ki}-{\hat{H}}_{ki}\right|}^{2}$$

where $H_{k}$ is the desired or target output and ${\hat{H}}_{k}$ is the actual output vectors of the network model for a training data set on pattern k, and i refers to the ith output neuron. The optimization algorithms will be applied to handle the learning process in the ANN model and to achieve the best performance by solving Equation (3). However, these traditional methods need to simultaneously satisfy smoothness, continuity and differentiability criterion of the objective function (error evolution method) to find the local optimal weights and parameters in ANN. In general, these traditional algorithms are efficiently limited in solving high level of uncertainties or stochastic application nature.

Therefore, it is important to examine new optimization algorithms such as the GROM technique to efficiently handle the uncertainties in EFWS, such as weather and water flow data, and increase the forecast accuracy. In this article, the GROM algorithm is used as a new metaheuristic optimizer to train the ANN to achieve global parameters and weights within a minimum computational time. The GROM is used a growth searching patterns nature, namely the golden ratio, was presented by Fibonacci in [25,33]. The growth searching in GROM angle and magnitude are determined based on the golden ratio, as described by Equation (4).

(4) $${\hat{H}}_{new}={\hat{H}}_{old}+r\;\left(\frac{1}{GR}\right)\;\left({\hat{H}}_{best}-{\hat{H}}_{worst}\right)$$

where *r* is a random value and *GR* is the golden ratio [25]. The use of the GROM aims to improve the searching technique and find the optimal solution with lower computational cost. The ANN forecast model based on GROM procedure is presented in Table 3. In addition, the GROM technique is suitable for complex and timely problems where it does not include any tuning steps. This helps to minimize the convergence rate and the running simulation time (computational cost), especially the EFWS working with 10 min time resolution. In this paper, the GROM model parameters have been optimally selected over a wide range of values, as in [33]. In general, there are many optimization algorithms such as Adam optimization algorithm and Root Mean Square propagation (RMSProp) have been used in solving stochastic objective functions in ANN. However, the Adam algorithm is mainly employed with deep learning process and showed better results compared to RMSProp but with high computational cost. There are many heuristic optimization algorithms as part of modern optimization such as GROM algorithm has been recently used for different engineering problem. For example, GROM evaluated in [25,33] by using different 35 benchmark test problems outperformed a number of well-known optimization algorithms. The employing of modern heuristic optimization algorithms such as GROM are a consideration of this work; there are limited number of studies that consider the benefit of using metaheuristic optimization algorithms. Where no optimization algorithm can effectively solve all the optimization problems, it is significant to employ different methods with different applications. For example, the results in [32,33] showed that there is a difficulty for a specific optimization to a suitable solver for all application and ANN models. This gives a motivation to use new metaheuristic optimization method, GROM, algorithm with ANN where it can be beneficial for imprinting the accuracy of the ANN forecast model for EFWS with high uncertainty. In addition, the studies on furcating the water level or flash flood by using new optimization problems are sparse in the literature and there are no studies using the GROM.

### 4.2. The Forecast Model Evaluation

In this section, the results of forecast models in EFWS is presented and discussed. In order to evaluate the performance of these models, the Mean Absolute Percentage Error (MAPE) and Root Mean Square Error (RMSE) are used, as shown in Equations (5) and (6) [24,30].

(5) $$MAPE=\frac{100}{T}\;\overset{T}{\underset{t=1}{\sum }}\left|\frac{H\left(t\right)-\hat{H}\left(t\right)}{H\left(t\right)}\right|,$$

(6) $$RMSE=\sqrt{\frac{\sum_{t=1}^{T}{\left(H\left(t\right)-\hat{H}\left(t\right)\right)}^{2}}{T}}$$

where H(t) is the actual water level at current time step t, $\hat{H}\left(t\right)$ is the forecasted water level; T is the total number of observations (time steps). The MAPE and RMSE are two of the most standard and common forecast evaluation methods, where MAPE is a scale-independency approach and easy to interpret and RMSE shows the overall value of the error. Throughout Section 5, the following is discussed:

- Evaluating different exogenous variables and ANN structure parameters to determine the optimal parameters for the ANN models
- Overall comparisons are presented for the traditional ANN and ANN optimized by GROM.
- Evaluating the significance of using the rolling forecasts compared to a fixed model.

In Section 5, the overall MAPE is presented the MAPE for data set points from the first point until the end of data set. The daily MAPE is calculated as the average of MAPE values for each day data set points from first point in the day until the end of the same day.

## 5. Results and Discussion

This paper aims to develop and present an efficient and sustainable early warning system for flash flood forecast in Jordan by creating a warning plan for a day ahead to minimize the flood risks. The real-time measurements from ultrasonic sensor and weather forecast data are fed to the rolling forecast model to overcome some of the forecast error challenges such as a misleading warning alarm. The EFWS was developed based on fixed and rolling forecast models to investigate the significance of using the updated data and real-time measurement system in the forecast model to improve the forecast models’ performance. The fixed and rolling forecast models for traditional ANN and ANN with the GROM method will be compared and discussed in this section. Furthermore, the ANN forecasts for EFWS application are evaluated for different parameters and structures, such as the number of layers, to choose the optimal and suitable model parameters. The impact of these parameters on the forecast model accuracy is examined.

### 5.1. Risk Knowledge and Flood Warning System

The unplanned urban encroachment in Jordan, in particular downtown/Amman, have experienced flash flood events in 2017, 2018 and 2019 due to the city nature and climate change [8,9]. These flood events caused a loss in human life, properties and economic loss. The repetition of these flash flood events highlights the needs to develop an efficient and accurate early flood warning system to protect the community and city from any future flash floods events. The proposed EFWS will have a direct impact on minimizing flood risks in different terms (economic, infrastructure damages, human lives, energy consumption, environmental burden and public health). In this project, the EFWS aims to generate a warning plan for one day ahead based on a rolling flood forecasting profile. This will increase the public awareness time and increase the efficiency of the warning model.

One of the main results in this paper was defining and determining the water risk level during different weather and water flow scenarios. The warning model is developed based on threshold values of the water level in the drainage channel. The risk thresholds were determined based on statistical analysis for the water flow in the drainage system. The flash flood warning scenarios were mapped, as shown in Figure 10. The green level is when *H* is at less than 25% of the capacity of the drainage channel, the yellow level is between 25% and 50% of the capacity, the orange level is between 50% and 75% of the capacity and the red level if it exceeds 75% of its capacity. In this work, a day-ahead warning plan will be issued and updated every 10 min based on real-time measurements. The time between the alert and potential flash with a minimum value equal to 10 min is enough for people to exit the risk zone. The EFWS is designed and developed to issue one day ahead alert and at every time step the alert plan will be updated. The proposed EFWS will generate different type of notifications if the water level estimated in the orange and red levels, as shown in Figure 11. The following notifications and EFWS reports will automatically change according to the updated water level estimation.

- The EFWS generates daily and hourly reports (excel sheets) and dialog box warning (lights and sound) in the workstation (control center). This data and information will be available for the decision-maker and government to make necessary decisions and actions.
- The EFWS model will send an email notification to selected emails by control center holder such as private and government stockholders and emergency team.
- To increase the awareness of the flash flood risk among communities, the EFWS model will post a warning message on Twitter if the estimated water level in orange or red zones.

### 5.2. Effect of Exogenous Variables and Model Parameters on Forecast Models

As discussed in Section 4.1, there is a lack of investigation about the impact of exogenous input variables and model parameters for the ANN forecast model for flash flood or water level events. This investigation aims to optimally determine the ANN structure (input, hidden, and output layers). There is no favorite way to guarantee the optimal structure for the ANN forecast model, and usually it depends on the availability of data, computational cost, correlations between the inputs and outputs and complexity of the problem. In general, the optimal structure of the ANN model is usually determined based on the trade-off between the computational cost and model accuracy and avoiding the overfitting situation. The analysis in this section used the training and validation data sets to investigate the forecast model accuracy with different ANN structural for EFWS. The experiment in this section was carried out to select the optimal number of hidden layers, number of neurons and input variables for EFWS. Firstly, we developed a reference traditional ANN forecast model (namely: Model A) based in the literature [24,30,31] with the following parameters:

- Input exogenous variables: adding water level at time *t*, $\Delta \hat{L}$ (*t*), and real-time measurement for the water level, L (t−10)
- Hidden layers: two layers.
- Hidden neurons in each layer: 10 neurons.
- Rolling forecasts.

In the following, the reference model, Model A, performance result is presented and compared to other ANN models by changing a single parameter at every trial, to show the impact of each parameter and select the optimal one using the training and validation data sets.

The number of hidden layers: Model A is tested by changing the number of hidden layers between 1 to 5 layers, in order to avoid overfitting and high computational costs with high number of layer systems. The overall and daily MAPE results over the training period is presented in Table 4. The result shows that Model A with two hidden layers achieved the minimum MAPE of 1.4% and the lower accuracy model was the model of 5 layers with 6.7%. In general, all models with different hidden layers showed MAPE results under 7%. In addition, the average daily MAPE for model A outperformed other models and scored MAPE equal to 1.3%. As a result, the optimal number of hidden layers for the EFWS and this data set is two hidden layers.

The number of neurons in each hidden layers: the literature showed that less than 20 neurons at each layer can be sufficient to achieve the optimal results without leading to over-fitting in the ANN for different applications. Therefore, this section investigated the optimal ANN structure for EFWS with a different number of hidden neurons. Table 5 shows the mean of the daily MAPE results for the reference ANN model, Model A, with different hidden neurons from 10 to 50. The minimum mean of the daily MAPE value was 1.1% for 20 neurons at each hidden layer. In general, the MAPE values increased with increasing the number of hidden neurons and achieved 4.8% as a maximum mean of the daily MAPE values. Therefore, the optimal number of hidden neurons are 20 at each layer for the EFWS and this data set.

The input exogenous variables: the reference forecast model, Model A, with $\Delta \hat{L}\left(t\right)$ and L (t−10) as input exogenous variables are used to be compared to other models by adding new exogenous variables to the reference model. The new exogenous variables, ($A_{1},\;A_{2},\;A_{3},\;A_{4}$), which have been added to Model A are described in Table 6.

**Figure 14:**
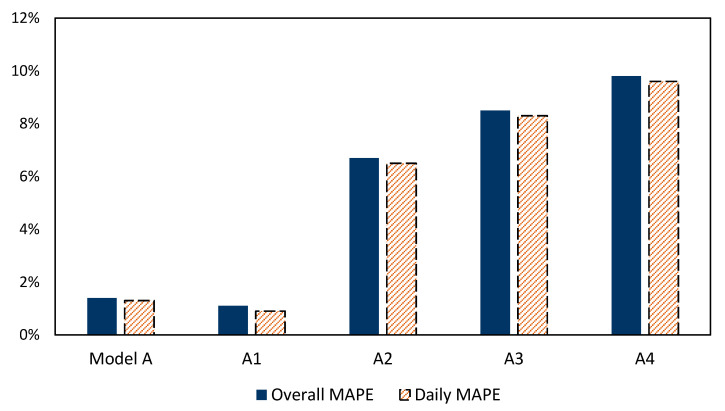
The overall and daily MAPE for ANN forecast model with input exogenous variables.

Figure 14 shows that adding $A_{1}$ exogenous variables to Model A improved the forecast model performance and achieved 0.9% daily MAPE. It is mainly related to the high relationship and correlation between current water level *H*(*t*) and these external variables which are associated more with the water level compared to other variables. On the other hand, the input exogenous variables from $A_{2}$ to $A_{4}$ had a high MAPE compared to Model A, $A_{1}$ and $A_{2}$. This is presented the exogenous variables $A_{2}$ to $A_{4}$ as misleading variables for the ANN forecast model. This indicates, in the EFWS data set, that the $A_{1}$ exogenous variables in line with the $\Delta \hat{L}$ (*t*) and *L* (*t* − 10) are recommended as inputs for the ANN forecast model. Model A and adding $A_{1}$ as exogenous variable performed similarly with an accuracy of less than 1.5%.

In order to investigate the correlation between the exogenous variables ($A_{1},\;A_{2},\;A_{3},\;A_{4}$) and the the water level *H*(*t*), Table 7 presents the R-squared values for the linear relationship between them. The R-squared is a statistical analysis tool that aims to show the correlation between variables. The water level of previous time steps from 20 min until one hour ($A_{1}$) showed a direct and high correlation with R-squared equal to 93.7%. The exogenous variables ($A_{2},\;A_{3},\;A_{4}$) showed a limited correlation compared to $A_{1}$ with R-squared below 52 %. Therefore, using of $A_{1}$ with Model A showed most accurate results compared to other exogenous variables as shown in Figure 14.

### 5.3. Traditional ANN Model and ANN Forecast Model Optimized by GROM

Firstly, the optimal structure of the ANN forecast model (both traditional and GROM) which will be used in the EFWS has been determined based on the experiment results from the previous section—as follows:

- Input exogenous variables: adding water level at time *t*, $\Delta \hat{L}$ (*t*), and real-time measurement for the water level, L(t−10) and $A_{1}$ (the previous time steps from 20 min until one hour)
- Hidden layers: two layers.
- Hidden neurons in each layer: 20 neurons.

Then, the MAPE scores were calculated over the testing period for the traditional ANN model and ANN model optimized by GROM using the testing data sets. Table 8 shows the overall and mean of daily MAPE scores for the traditional and ANN model optimized by GROM. In terms of accuracy, the ANN model optimized by GROM outperformed the traditional ANN model for the overall or daily results over the testing period. The overall and daily MAPE scores for the ANN model optimized by GROM were improved by 40% and 37.5%, respectively, compared to the traditional ANN model. This is mainly related to the ability of the GROM method to solve complex problems with low computational cost. In this work, the percentage of improvement or reduction is calculated based on the formula in Equation (7) [24,30].

(7) $$\mathrm{Improvement}\%=\frac{\mathrm{final}\;\mathrm{value}-\mathrm{starting}\;\mathrm{value}}{\mathrm{starting}\;\mathrm{value}}100,$$

Furthermore, this section presents a comprehensive analysis of different common and recent metaheuristic algorithms from the literature, namely: Modified Particle Swarm Optimization (MPSO) and Teaching–Learning-Based Optimization (TLBO) [32,33]. In Table 8, the ANN forecast model equipped with metaheuristic optimization algorithms (GROM, MPSO and TBLO) outperformed the traditional ANN algorithm. Furthermore, the ANN optimized by GROM scored the best ANN forecast accuracy compared to all forecast model with new optimization algorithms (TLBO and MPSO).

### 5.4. Evaluating the Significance of Using the Rolling Forecasts Compared to a Fixed Model

**Figure 15:**
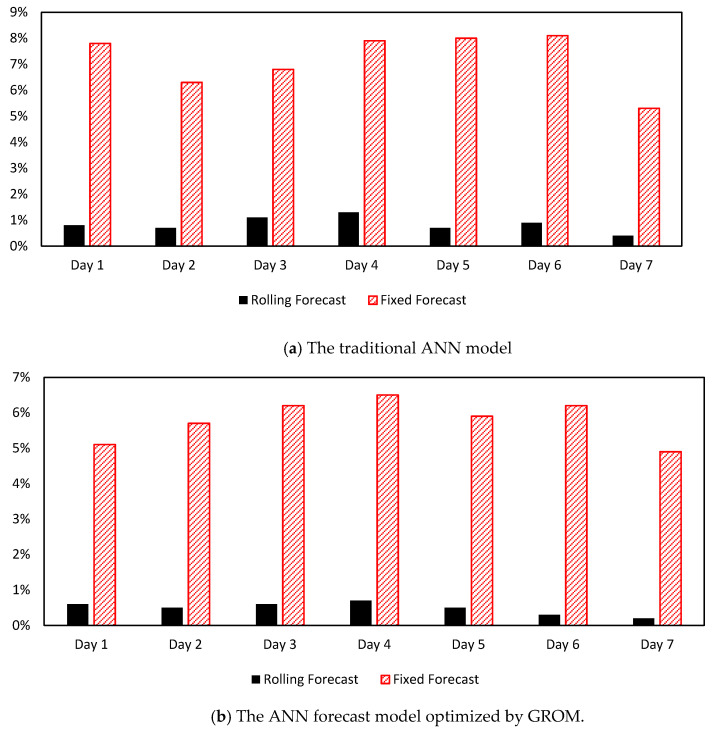
The daily MAPE scores for rolling and fixed forecasts models.

In this section, the ANN forecast models both traditional and GROM are extended and modified to create a rolling forecast in EFWS. The rolling process aims to generate a water level prediction profile for one day ahead with 10 min time resolution, and then the model will be updated every time step by using the real-time measurement and forecast error, as shown in Figure 12. The rolling process will help to minimize the impact uncertainties and improve the forecast model accuracy compared to a fixed forecast model using the testing data sets. The fixed model aims to forecast the water level over one day ahead without any updating for the data or model during the daytime. Figure 15 presents the daily (seven days) MAPE scores for the fixed and rolling forecast model. The comparison results showed that the performance of the rolling forecast models (traditional and ANN optimized by GROM) significantly improved the MAPE scores compared to the fixed forecast model. For instance, on Day 2 the MAPE score decreased from 6.3% to 0.7% for traditional ANN and from 5.7% to 0.5% for the ANN model optimized by GROM. The maximum improvements in the daily MAPE score were on Day 6 by 93.5% for the GROM model and 92.4% for the traditional ANN model on Day 7. These results indicate that the rolling forecast (updating the model each time step) will increase the forecast model accuracy. On the other hand, the rolling process will require real-time measurement and updating the model parameters at each time step, which will increase the computational costs compared to fixed forecast.

### 5.5. Discussion

Flash flood and water level forecasting has become a significant tool for the community. The importance of an effective and accurate forecast model is to minimize utility risks. Because there is limited research on flash flood and no research discussing the optimal forecast model parameters with this kind of application, this paper presented different forecast models—traditional ANN and ANN with GROM. In this research, a number of models have been implemented and tested to forecast the water level over a day ahead. After the weather data, location data and exogenous variables were analyzed, we examined different options for forecast model inputs. In this paper, each model has been trained separately using the training data set of historical data. The evaluation method results for each model, which has different input variables discussed in Section 5.2 showed that adding $A_{1}$ exogenous variables to Model A improved the forecast model performance and achieved 0.9% daily MAPE and outperformed all other models. The analysis of the results showed that it is not recommended to add $A_{2}$ to $A_{4}$ as exogenous variables in the ANN model. Moreover, the overall and mean of daily MAPE scores show that the ANN model optimized by GROM outperformed the traditional ANN model by improving the overall and daily MAPE scores for the ANN model optimized by the GROM by 40% and 37.5%, respectively, compared to the traditional ANN model. This is mainly related to the ability of the GROM method to solve complex problems with low computational cost. In addition, the rolling forecast process helped to improve the MAPE scores compared to the fixed forecast model. However, the rolling process needs more effort for tuning and a higher computational cost compared to the fixed forecast. The updated data process (rolling forecast) gives an improved reduction in the significant forecast error. The extra information that becomes available in each horizon step helps to improve the forecast model performance.

## 6. Conclusions

Recently, flash flood events occurred in the downtown/Amman area (2018, 2019 and 2020). Therefore, this study has presented a novel and efficient rolling EFWS for flash floods in streets (drainage system) using real-time measurement based on ultrasonic sensors and the ANN model. The proposed system has developed based on two main parameters. Firstly, real-time water level measurements through an ultrasonic sensor, located in the street water drainage. Secondly, a rolling flood forecasting model based on ANN and GROM algorithms to estimate the water level in the water drainage systems during the storm period. Unlike previous studies, this work has presented an investigation to determine the optimal structure of the ANN model for flood application. In addition, the analysis of the results showed the significance of using the rolling forecast model compared to fixed models by improving the accuracy by 93.5%. The GROM algorithm helped the ANN to handle uncertainty and increased the forecast model accuracy by 40% compared to the traditional ANN. Finally, the implementing and developing internet-of-things (IoT) concept for a network of sensors in EFWS over the country will part of our future work.

## Figures and Tables

**Table 1:** The equipment specifications for the real-time monitoring system.

Equipment	Specifications
Arduino microcontroller (ATMega328)	Input: Voltage: 5~12 V, current: 50 mA, Temp range: −40 °C to 80 °C
Ultrasonic Sensor (HC-SR04)	Input: Voltage: 3.3~5 V, Distance range: 2 cm–400 cm
GPRS/3G/4G module	Input power: Voltage: 5 V, Output: SMS texts
Solar panel	O/P: 6 V, Power: 3 W, Current: 300 mA
Battery lead acid	Voltage: 6 V, Cpacity: 12 Ah.

**Table 2:** The rolling forecast model procedures.

Step	Description
1	Estimate the 10 min adding water level, $\Delta \hat{L}$, for one day ahead using the weather forecast data, the water drainage flow and maximum discharge of the drainage channel data.
2	Collect the real-time water level measurements L at time (t−10).
3	Generate a flash flood (water level) prediction profile), $\hat{H}$, for one day ahead with 10 min resolution by ANN forecast model. The details of forecast model will be discussed in Section 4.1.
4	Collect and feed all updated data (water level measurements and forecast error) back to the forecast model to reforecast the water level.

**Table 3:** The GROM learning procedure in the ANN forecast model.

Step	Description
1	Create an initialization population for the learning parameters in the ANN forecast model.
2	Calculate the mean value of the population.
3	Calculate the objective function (forecast error), as fitness term, for all and mean populations. To achieve convergence within minimum time, if the fitness value of the mean solution is better than the worst solution, the worst solution will be replaced by the mean solution.
4	Create a random solution vector in the population to determine the new searching step angle and movement using the golden ratio method. This process aims to move the searching towards the best solution area.
5	Compare the fitness value of the new random solution and the mean solution. This comparison aims to create a random searching and increase the ability of to search the whole solution of the fitness function and achieve the global solution.
6	The optimal solution for the ANN parameters will achieve the minimum fitness value.

**Table 4:** The overall and daily MAPE forecast error for the traditional ANN with a different number of hidden layers.

Number of Hidden Layers	Overall MAPE	Mean of the Daily MAPE
1	3.2%	3.0%
**2** (Model A)	**1.4%**	**1.3%**
3	4.5%	4.4%
4	5.2%	5.1%
5	6.7%	6.6%

**Table 5:** The mean of the daily MAPE values for Model A with different numbers of neurons.

Number of Neurons		First Hidden Layer				
		10	20	30	40	50
**Second hidden layer**	10	1.3%	1.5%	2.2%	2.9%	3.3%
	**20**	1.2%	**1.1%**	2.3%	2.2%	3.1%
	30	1.6%	1.9%	2.5%	3.4%	3.4%
	40	2.1%	1.6%	2.9%	3.7%	4.2%
	50	2.3%	1.9%	2.7%	3.9%	4.8%

**Table 6:** The exogenous variables with the ANN forecast model (Model A).

	$A_{1}$	$A_{2}$	$A_{3}$	$A_{4}$
Description	the water level of previous time steps from 20 min until one hour	the previous day water level at the same time	the previous day data of daily rain intensity	the hourly temperature
Unit	cm	cm	mm/hour	°C

**Table 7:** The R-squared values for the relationship between *H*(*t*) and ($A_{1},\;A_{2},\;A_{3},\;A_{4}$).

Correlated Variables	$R^{2}$
$H(t)\;\mathrm{vs}.\;A_{1}$	93.7%
$H(t)\;\mathrm{vs}.\;A_{2}$	51.8%
$H(t)\;\mathrm{vs}.\;A_{3}$	35.1%
$H(t)\;\mathrm{vs}.\;A_{4}$	27.7%

**Table 8:** The MAPE scores the traditional ANN model and ANN forecast model optimized by GROM.

		Daily MAPE	Overall RMSE
Traditional ANN	1.0%	0.8%	17 cm
ANN optimized by GROM	0.6%	0.5%	7 cm
ANN optimized by MPSO	0.9%	0.8%	14 cm
ANN optimized by TLBO	0.7%	0.6%	10 cm

## Data Availability

Not available.
